# Bioaugmented Mixed Culture by *Clostridium aceticum* to Manipulate Volatile Fatty Acids Composition From the Fermentation of Cheese Production Wastewater

**DOI:** 10.3389/fmicb.2021.658494

**Published:** 2021-09-03

**Authors:** Merve Atasoy, Zeynep Cetecioglu

**Affiliations:** Department of Chemical Engineering, KTH Royal Institute of Technology, Stockholm, Sweden

**Keywords:** volatile fatty acid, acetic acid, *Clostridium aceticum*, bioaugmentation, bacterial community profile, qPCR, cheese production wastewater, fermentation

## Abstract

Production of targeted volatile fatty acid (VFA) composition by fermentation is a promising approach for upstream and post-stream VFA applications. In the current study, the bioaugmented mixed microbial culture by *Clostridium aceticum* was used to produce an acetic acid dominant VFA mixture. For this purpose, anaerobic sequencing batch reactors (bioaugmented and control) were operated under pH 10 and fed by cheese processing wastewater. The efficiency and stability of the bioaugmentation strategy were monitored using the production and composition of VFA, the quantity of *C. aceticum* (by qPCR), and bacterial community profile (16S rRNA Illumina Sequencing). The bioaugmented mixed culture significantly increased acetic acid concentration in the VFA mixture (from 1170 ± 18 to 122 ± 9 mgCOD/L) compared to the control reactor. Furthermore, the total VFA production (from 1254 ± 11 to 5493 ± 36 mgCOD/L) was also enhanced. Nevertheless, the bioaugmentation could not shift the propionic acid dominancy in the VFA mixture. The most significant effect of bioaugmentation on the bacterial community profile was seen in the relative abundance of the *Thermoanaerobacterales Family III*. *Incertae sedis*, its relative abundance increased simultaneously with the gene copy number of *C. aceticum* during bioaugmentation. These results suggest that there might be a syntropy between species of *Thermoanaerobacterales Family III*. *Incertae sedis* and *C. aceticum.* The cycle analysis showed that 6 h (instead of 24 h) was adequate retention time to achieve the same acetic acid and total VFA production efficiency. Biobased acetic acid production is widely applicable and economically competitive with petroleum-based production, and this study has the potential to enable a new approach as produced acetic acid dominant VFA can replace external carbon sources for different processes (such as denitrification) in WWTPs. In this way, the higher treatment efficiency for WWTPs can be obtained by recovered substrate from the waste streams that promote a circular economy approach.

## Introduction

The current size of global chemical production is unknown. In Europe, it was 330 million tons in 2007 (Eurostat, 2020), and is increasing by a 7% compound annual growth rate (UNEP, 2013) to meet the demands for industrial, agricultural, pharmaceutical applications. On the other hand, the adverse environmental effects of petroleum based production have resulted in an increase of CO_2_ emissions from two billion tons to over 36 billion tons in the last 115 years (Ritchie and Roser, 2019). Therefore, sustainable, environmentally friendly, and economically competitive bioproduction is a vital method in achieving Sustainable Development Goals (United Nations, 2018) and the targets of the Paris Agreement, (UNFCCC, 2015). One of the crucial methods of attaining sustainable bioproduction is transforming traditional wastewater treatment plants into biorefineries, that use waste streams as feedstock (Dietrich et al., 2017; Mussatto, 2017).

Several studies for biobased chemical production from waste streams have been conducted in recent years (Lu et al., 2019; Xie et al., 2019). Foremost among them, volatile fatty acid (VFA) is one of the most promising products because of its versatile usage area in post stream and upstream applications (Khan et al., 2016b; Atasoy et al., 2018). Nevertheless, low production efficiency, unstable VFA composition for upstream and post stream applications, complications in purification, and separation of the end products, post-process requirements, and high substrate cost, etc., limit the industrialisation of biobased VFA production. Various studies have been carried out to enhance biobased VFA production efficiency by optimizing operational and environmental conditions (Khan et al., 2016a; Bhatia and Yang, 2017; Atasoy et al., 2018; Fang et al., 2020). These studies showed that different parameters such as pH, temperature, retention time, loading rate, reactor type, and mixing, etc., must be taken into account for efficient biobased VFA production.

The end product spectrum of VFA is another crucial factor in post stream and upstream applications. For example, acetic acid dominant VFA as a carbon source achieved the highest polyhydroxyalkanoates (PHA) production yield (Ciesielski and Przybylek, 2014; Kedia et al., 2014) and acetic acid has been used as an efficient carbon source for denitrification processes (Du et al., 2019). Furthermore, every industry has different feedstock requirements, for example, acetic acid has been extensively used in the polymer industry for the production of vinyl acetate monomer (Gunjan and Haresh, 2020). Thus, enhancement of specific acid concentration in the VFA mixture is preferred for easier separation/purification of the end product. For these reasons, the efficient and sustainable production of the desired acid composition is one of the primary research problems that need to be addressed in the commercialization of biobased VFA production. Therefore, the current study aimed to develop acetic acid dominant VFA mixture production.

Acetic acid comprises a large part of the VFAs market (Bhatia and Yang, 2017) and has been used as a vinyl acetate monomer, purified terephthalic acid, acetate esters, acetic anhydride in several industries such as chemical, food and beverage, inks, paints, and coatings, etc., (Vidra and Németh, 2018; Allied Market Research, 2020). The global acetic acid market is estimated to reach 20.3 million tons by 2024 (Expert Market Research, 2019). Nevertheless, more than 90% of acetic acid is produced synthetically (Atasoy et al., 2018). In several wastewater treatment plants, acetic acid has been used as a carbon source in the denitrification process (Mielcarek et al., 2018; Du et al., 2019). However, this acetic acid is mostly bought externally, which is obtained by petroleum-based methods. Furthermore, biobased acetic acid is approved and generally recognized as safe (GRAS) by the United States Food and Drug Administration (FDA, 1989) and has been preferred as a food additive (Younes et al., 2020). In this sense, biobased acetic acid production is essential to achieve sustainable and environmentally friendly chemical production. Accordingly, the current study aimed to produce an acetic acid dominant VFA mixture by the application of a bioaugmentation strategy.

Recent studies suggested that bioaugmentation is a successful strategy to not only enhance microbial community performance for obtaining desired products (Tang et al., 2019; Atasoy and Cetecioglu, 2020; Li et al., 2020; Wu et al., 2020) but also improve the microbial community and their interactions for better adaptations to various environmental conditions (Tabatabaei et al., 2020). Bioaugmentation is a promising approach by adding microorganisms externally to the existing microbial community for improving the degradation rate of the contaminants (Cirne et al., 2006), enhancing the production efficiency of specific products (Chi et al., 2018; Tabatabaei et al., 2020), reducing the inhibition effects of some substances in the process (Yang et al., 2019), which has been used to find a solution for several practical issues in wastewater treatment plants (Herrero and Stuckey, 2015; Hong et al., 2020). Yang et al. (2019) investigated the effects of bioaugmentation with several pure cultures on anaerobic digestion to improve biogas production *via* preventing ammonia inhibition (Yang et al., 2019). In anaerobic digestion, the microbial community is comprised of undefined mixed culture, which is robust and easy for operation; nevertheless, monoculture produces a specific product. Therefore, bioaugmentation is a promising strategy for developing a new microbial consortium for strong, higher productivity and open for manipulation to produce targeted product profiles.

*Clostridium aceticum* is the first isolated acetogen from soil, found by Wieringa in 1936 (Küsel and Drake, 2011). It is a well-known acetic acid producer bacteria (Braun et al., 1981). *C. aceticum* is strictly anaerobic and can grow both autotrophically and heterotopically (Poehlein et al., 2015). Because of its versatile growth ability, *C. aceticum* has been widely used for acetic acid production (Sim et al., 2007; Arslan et al., 2019; Riegler et al., 2019). In our previous study, we used *C. aceticum* for acetic acid production from semi-synthetic milk processing wastewater fermentation under alkali pH (Atasoy et al., 2020a). Therefore, *C. aceticum* is selected as pure culture in the current study to bioaugment mixed microbial culture for acetic acid dominant VFA mixture.

Although different types of waste streams have been used for acetic acid production *via* fermentation, one of the most promising waste streams comes from dairy industry, which has a massive wastewater production volume (Hansen, 1974) with a rich carbohydrate, protein, and fat content (Britz and van Schalkwyk, 2006). Lagoa-Costa et al. (2020) showed that cheese whey has great potential for VFA production as a substrate because it includes more than 90% of easily degradable compounds. Their results stated that acetic acid concentration in VFA mixture from cheese whey fermentation at acidogenic pH was almost similar under different retention times, food/microorganism (F/M) ratios, and sludge retention times (SRTs), despite the degree of acidification and the acidification yield was changed under different conditions. On the other hand, Jankowska et al. (2017) showed the effects of various pH on VFA composition from cheese whey fermentation: acetic acid production was around 20% under acidic pH and approximately 40% without pH adjustment (neutral pH), whereas, it was more than 91% under alkali pH (Jankowska et al., 2017). Atasoy et al. (2019a) stated that higher acetic acid concentration was obtained from the monoculture (*C. aceticum*) (743 mg COD/L) than mixed culture (541 mg COD/L) from milk processing wastewater fermentation under alkali pH (Atasoy et al., 2019a). As previously stated, various waste streams as substrate provide an excellent opportunity for acetic acid and VFA production *via* fermentation. Nevertheless, further studies are required to increase the production yield for economically competitive and sustainable VFA production from waste streams.

In the current study, enhancement of acetic acid in VFA mixture was aimed by bioaugmentation. With this approach, the produced acetic acid dominant VFA mixture could be used in different processes (such as denitrification) as a carbon source to achieve a circular economy approach in WWTPs. In this regard, the mixed culture is bioaugmented by *C. aceticum.* Cheese production wastewater was used as a substrate to integrate the bioaugmentation strategy into industrial waste streams. The long term ASBR was operated under alkali pH to investigate the effects of the bioaugmentation. The bacterial community profile was analyzed by 16S rRNA sequencing and *C. aceticum* was quantified by quantitative real-time polymerase chain reaction (qPCR). Cycle analysis was also conducted to investigate the effects of bioaugmentation on acid composition.

## Materials and Methods

### Substrate and Inoculum

Cheese production wastewater was used as a substrate in all reactors. The wastewater was taken from the cheese production industry, which is located in Sweden. The wastewater included 20000 ± 60 mg COD/L, 200 mg/L total nitrogen, 18 mg/L total phosphate, and 11 mg/L orthophosphate. The wastewater contained 14.26 ± 6 mg COD/L VFA and 0.22 ± 0.08 mg COD/L lactic acid. The medium, vitamin, and trace element solutions were prepared according to the OECD 311 (OECD, 2003) and used for pure culture incubation. The medium and trace element solution was autoclaved separately for 30 min at 121°C. The vitamin solution was also filtered through a membrane filter with a 0.22 μm pore size filter for sterilization.

#### Mixed Culture

The granular seed sludge (≈3.5 mm with 43% total solids (TS) and volatile solids (VS) 30%) were used as a mixed culture, which was collected from the UASB reactor at Hammarby Sjöstadsverk Pilot Plant, Stockholm, Sweden. It was stored at +4°C until the experiments were set up. The detailed characterization of granular seed sludge was described by Atasoy et al. (2019b).

#### Monoculture

The bioaugmentation of mixed culture was applied by *C. aceticum* (No. 1496, DSMZ, Germany) to enhance acetic acid production in mixed culture fermentation. The monoculture was grown in YPD Liquid Media (VWR Life Science, Sweden) at 32.5°C in an incubator with 120 rpm mixing for 36 h under anaerobic conditions. Before bioaugmentation, the monoculture was cultivated with the substrate to observe and evaluate their growth and interactions. The growth of monoculture was observed by using OD600. The pure culture from the actively growing culture (the OD600 was 2.0) was used for bioaugmentation. In addition, approximately 50 mL (aliquoted 5 mL) of pure culture was stored in glycerol (50% as volume) at −80°C for further usage.

### Reactor Design and Operation

**FIGURE 1:**
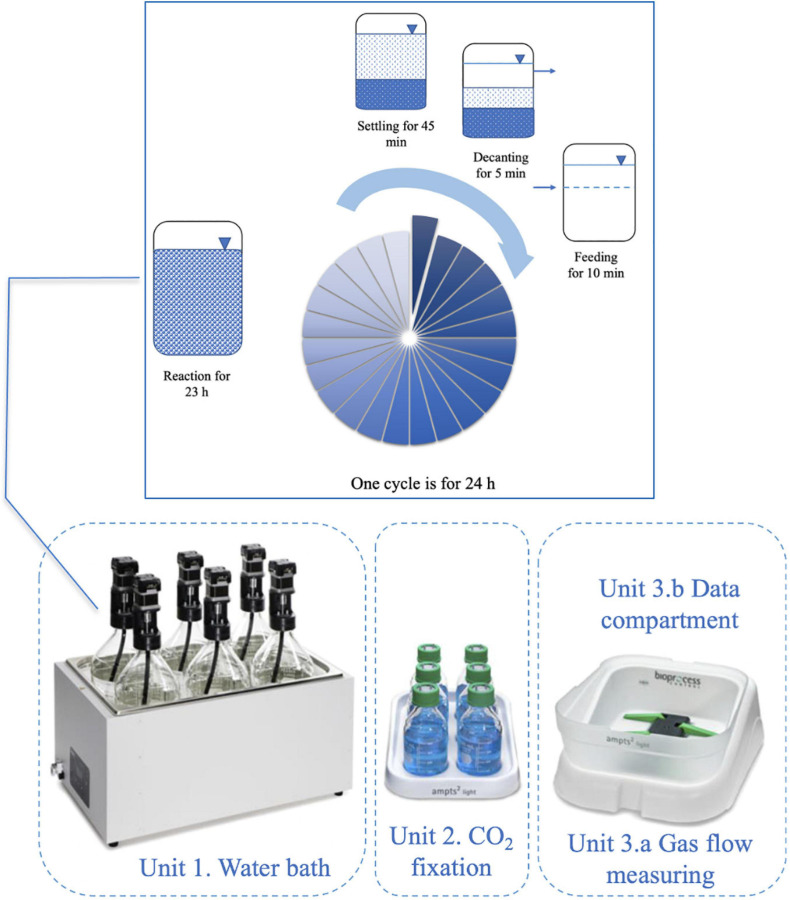
The AMPTS II System (Bioprocess Control, Sweden) for anaerobic sequencing batch reactors and detailed operation regime.

The AMPTS II System (Bioprocess Control, Sweden) was used for an anaerobic sequencing batch reactor which had 1400 mL active volume, 2000 mL total volume. The reactors were operated by cycling through a sequence of four phases in a single reaction vessel, the detailed operation of the reactors was presented in Figure 1. The system was mixed continuously at 120 rpm under 35°C and pH 10 ± 0.5, which was adjusted using 2 M NaOH.

The reactors were fed in a stepwise manner with different organic loading rates (OLR) which are explained in detail in Atasoy et al. (2020a). In steady-state conditions (based on influent and effluent chemical oxygen demand (COD) concentrations), the bioaugmentation was applied with a 0.6 F/M ratio. The SRT was 35 days, calculated according to the VS loss during decanting. The hydraulic retention time (HRT) was 3.5 days.

#### Cycle Analysis

The cycle analysis was conducted to observe the bioaugmentation effects on VFA composition shift during a cycle, which was carried out after 7 days the bioaugmentation was completed in each reactor. The samples were taken in each hour during one cycle (24 h) as well as before (−1st hour) and after feeding (0th hour).

#### Bioaugmentation Strategy

The bioaugmentation strategy was applied as explained by Atasoy and Cetecioglu (2020). It included three main phases; Phase A: before bioaugmentation (steady-state conditions based on COD concentration), Phase B: during bioaugmentation (the monoculture was added to the bioaugmented reactor in each cycle for 7 days as 10% of the reactor volume), and Phase C: after bioaugmentation (the reactors were operated for two sludge ages to observe the growth of monoculture in mixed culture).

### Analytical Methods

During the whole operation, Total COD (TCOD), soluble COD (SCOD), organic acids, VFA compositions, and pH were monitored in both influent and effluent of the reactor. The COD equivalent of each VFA was calculated to validate the mass balances derived. The SCOD/TCOD, organic acids, total Nitrogen, and Phosphorus were measured using LCK 514 COD (100–2000 mg/L), organic acids LCK 365 Organic Acids (50–2500 mg/L), LCK 238 total Nitrogen (5–40 mg/L TN), and LCK 348 Phosphate (Orto + total) (0.5–5 mg/LPO4 – P) (Hach Lange, United States) cuvette tests by Hach Lange DR 3900 spectrophotometer. Also, TS and VS of the sludge were measured according to the Standard Methods (APHA et al., 2012). The concentration and composition of VFA (formic, acetic, butyric, propionic, valeric, isovaleric, hexanoic, and heptanoic acids) in the effluents were analyzed by gas chromatography (GC 6890, Agilent) with a flame ionization detector, as described in the previous study (Atasoy et al., 2019b). Biomethane production (BMP) was monitored during operation, nevertheless, a negligible amount of biomethane was produced because of alkali pH. Therefore, the biomethane data did not present in the results. During and after the feeding of the reactors, the reactors flushed with nitrogen gas to enable anaerobic conditions.

### Bacterial Community Analysis

The bacterial community profile was analyzed by 16S rRNA gene sequencing. Total genomic DNA from 0.5 g samples with three replicates were isolated using NucleoSpin Soil Kit, (Macherey-Nagel, Germany) following the manufacturer’s instructions. The bacterial 16S rRNA gene was amplified using primers 516F (5′ to 3′: TGC CAG CAG CCG CGG TAA) and 806R (5′ to 3′: GGA CTA CHV GGG TWT CTA AAT) (Caporaso et al., 2011; Klindworth et al., 2013). The PCR amplification conditions, purification and quantification of the PCR products, and preparation of the sequencing libraries were followed by Atasoy et al. (2019b). The samples were sequenced using the Illumina MiSeq platform by Science for Life Laboratory, the National Genomics Infrastructure, NGI (Sweden). Biophyton 1.78 was used to merge and quality filter the sequence data as well as to assign taxonomies at 97% similarity cut-off value (Cock et al., 2009). Raw sequence data is available at NCBI (project no. of PRJNA667606).

### Quantification of *Clostridium aceticum*

The *C. aceticum* was quantified by using quantitative real-time polymerase chain reaction (qPCR) by using total genomic DNA. For the DNA extraction, 0.5 g samples as triplicate were isolated by using NucleoSpin Soil Kit, (Macherey-Nagel, Germany). The concentration of the extracted DNA was measured by fluorimetry using Qubit dsDNA HS Assay Kit (Invitrogen, Thermo Fisher Scientific, North America).

Quantitative real-time polymerase chain reaction was performed by using Applied Biosystems^®^ QuantStudio^®^ 3 Real-Time PCR System Thermo Fisher Scientific (United States). For each PCR run with PowerUp SYBR Green Master Mix, Applied Biosystems (Thermo Fisher Scientific Co., United States) detection, a melting curve analysis was performed to confirm the specificity in each reaction tube by the absence of primer-dimers and other nonspecific products. Reactions for all samples were shown to have only one melting peak, which indicated a specific amplification, making it suitable for accurate quantification. Controls were included for each PCR run during the analyses. At the end of the reactions, melting curve analyses were applied to confirm the absence of primer dimers and nonspecific products.

The primer for the quantification of *C. aceticum* sets targeting formyltetrahydrofolate synthetase gene (fhs) fhs1 (GTW TGG GCW AAR GGY GGM GAA GG) and fhs2 (GAR GAY GGW TTT GAY ATY AC) (Xu et al., 2009). Although fhs gene encoding 10-formylte- tetrahydrofolate synthetase is used for quantification of authentic acetogenic bacteria (De Vrieze and Verstraete, 2016), it was used to quantify *C. aceticum* in the current study, since the bioaugmentation was applied by using a specific strain. Standard curves were obtained for QPCR constructed from PCR products of *C. aceticum* by using a 10-fold dilution series, separately. Standard curves were constructed in each PCR run, and the copy numbers of the genes in each sample were interpolated using these standard curves.

### Calculation of Products Yield

Acetic acid production yield (Y_acetic_) (Eq. 1) and total VFA production yield (Y_*VFA*_) (Eq. 2) were calculated as the ratio of acid concentration to the consumed COD concentration (Jankowska et al., 2017; Atasoy et al., 2020a).

(1) $$Y_{a\,c\,e\,t\,i\,c}=\frac{C_{a\,c\,e\,t\,i\,c}}{C_{C\,O\,D\,c\,o\,n\,s\,u\,m\,e\,d}}$$

(2) $$Y_{V\,F\,A}=\frac{C_{V\,F\,A}}{C_{C\,O\,D\,c\,o\,n\,s\,u\,m\,e\,d}}$$

where, C_acetic_ is the acetic acid concentration (g COD/L) in the effluent, C_VFA_ is the total VFA concentration (g COD/L) in the effluent and C_COD__consumed_ is the consumed COD concentration (g COD/L).

### Statistical Analysis

All experiments were conducted in triplicate, the standard deviation of the average results was calculated. Additionally, Pearson’s correlation analysis was conducted to identify the relationship between the quantification of monocultures and each acid type production. All statistical analysis was performed using IBM SPSS Statistics, Version 25.0.

## Results and Discussion

The effects of bioaugmentation on both the acetic acid production efficiency and total VFA production and acid composition were evaluated, separately. The assessment of acetic acid production efficiency was performed based on acetic acid concentration and quantification of *C. aceticum*, before, during and after the bioaugmentation. Additionally, the quantification of *C. aceticum* was correlated with the concentration of each acid type to investigate the effects of bioaugmentation regarding VFA composition from the mixed culture fermentation. Also, the cycle analysis to observe the shift of acids type during a cycle (24-h) was conducted.

### Acetic Acid Dominant VFA Production

**FIGURE 2:**
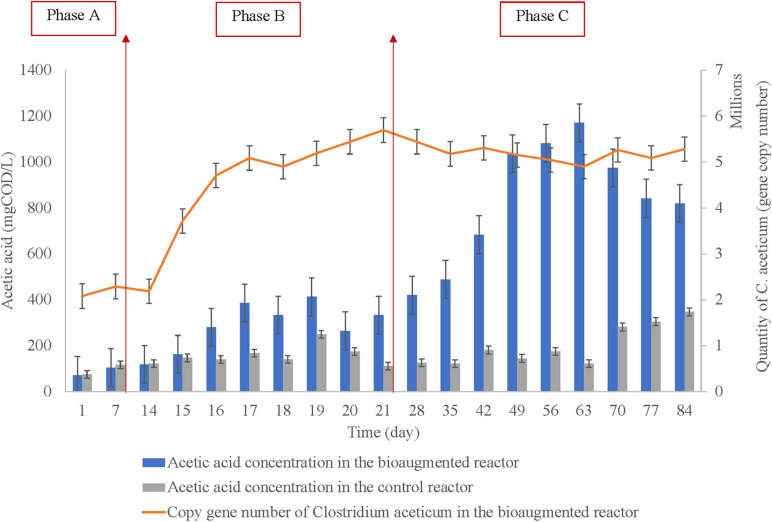
The acetic acid concentration in the control and the bioaugmented set and quantification of *Clostridium aceticum* in the bioaugmented set (Phase A: before bioaugmentation; Phase B: during bioaugmentation, and Phase C: after bioaugmentation).

The acetic acid concentration in the bioaugmented and the control reactor with the gene copy number of the *C. aceticum* is represented in Figure 2, regarding before bioaugmentation (Phase A), during bioaugmentation (Phase B), and after bioaugmentation (Phase C). The average acetic acid concentration before bioaugmentation (Phase A) was 88 ± 23 mg COD/L in the bioaugmented reactor, while it was 96 ± 29 mg COD/L in the control reactor. During bioaugmentation (Phase B), the concentration was 287 ± 102 mg COD/L in the bioaugmented reactor, 156 ± 43 mg COD/L in the control reactor. After bioaugmentation was completed, the acetic acid concentration increased to 836 ± 261 mg COD/L in the bioaugmented reactor, while it was 200 ± 87 mg COD/L in the control reactor. The results showed that the average acetic acid production was increased almost four times by bioaugmentation of *C. aceticum.* The maximum acetic acid production was 1170 ± 18 mg COD/L at day 63 in the bioaugmented reactor; it was 122 ± 9 mg COD/L in the control reactor. Based on the maximum acetic acid production, the concentration increased by almost 10 times in the bioaugmented reactor than in the control reactor.

The effects of bioaugmentation on acetic acid production were evaluated based on the literature studies as summarized in Table 1. For instance, Lagoa-Costa et al. (2020) used cheese whey for VFA production from acidogenic fermentation under different retention times and F/M ratios. In their study, the highest acetic acid concentration was obtained as 3580 mg COD/L from 55000 mg COD/L substrate concentration, regardless of operational parameters (Lagoa-Costa et al., 2020). In another study, the highest acetic acid concentration (5500 ± 70 mg COD/L) was obtained by lettuce fermentation (SCOD content of substrate was 35700 mg COD/L) under acidic pH (6 ± 0.4) from the evaluation of VFA production feasibility from agro-industrial waste (cucumber, tomato, and lettuce) (Greses et al., 2020). Lim et al. (2020) investigated VFA production from fermentation of palm oil mill effluent under pH 5 (Lim et al., 2020). Their results stated that the highest acetic acid concentration was obtained as 918 mg COD/L at day 30 from 33400 mg COD/L content of the substrate. She et al. (2020) used the pretreatment (freezing/thawing) for VFA production from waste activated sludge. The pretreatment increased the acetic acid production from 933 ± 46 to 1281 ± 57 mg COD/L as maximum concentration (the SCOD concentration of substrate was 5852 mg/L) (She et al., 2020). From the view of substrate concentration in terms of COD content, the bioaugmentation of *C. aceticum* enhanced acetic acid production *via* fermentation more than 10 times than other studies. Based on the calculation of acetic acid production yield, it was 0.05 gCOD/gCOD at Phase A, 0.125 gCOD/gCOD at Phase B, and 0.181 gCOD/gCOD at Phase C, respectively in the bioaugmented reactor with the 0.21 gCOD/gCOD (day 63) maximum value. Nonetheless, it was 0.06 ± 0.02 g COD/gCOD as an average at the control reactor in all phases. The bioaugmentation of *C. aceticum* increased acetic acid production yield 3.5 times than the control reactor. Jankowska et al. (2017) stated that the acetic acid production yield from cheese whey fermentation varied under different pH: as an average acetic acid production yield was 0.056 gCOD/gCOD under acidic pH, 0.31 gCOD/gCOD under alkali pH, and 0.164 gCOD/gCOD under neutral pH.

**TABLE 1:** Acetic acid production by mixed microbial culture fermentation under various operational and environmental conditions.

**Substrate**	**Operational and environmental conditions**	**Acetic acid production as concentration**	**Acetic acid production as yield**	**References**
Cheese production wastewater	Mixed culture fermentation under pH 10 at 35°C	After bioaugmentation (Phase C) 836 ± 261 mg COD/L as average; 1170 ± 18 mg COD/L as maximum in the bioaugmented reactor; and 150 ± 80 mg COD/L as average in the control reactor	After bioaugmentation (Phase C) 0.181 gCOD/gCOD as average; 0.21 gCOD/gCOD as maximum in the bioaugmented reactor; and 0.06 ± 0.02 gCOD/gCOD as average in the control reactor	This study
Cheese whey	Mixed culture fermentation under pH 5 at 30°C	The maximum acetic acid concentration 3580 mg COD/L	0.195 gCOD/gCOD	Lagoa-Costa et al., 2020
Agro-industrial waste: lettuce waste	Mixed culture fermentation under pH 6 ± 0.4 at 25°C	The maximum acetic acid concentration 5500 ± 70 mg COD/L	0.35 gCOD/gVS	Greses et al., 2020
Palm oil mill effluent	Mixed culture fermentation under pH 4.8–5.5 at 29°C	The maximum acetic acid concentration 918 mg COD/L	0.035 gCOD/gCOD	Lim et al., 2020
Waste activated sludge	Mixed culture fermentation under pH 6.8 at 35°C (application of freezing/thawing pretreatment)	The maximum acetic acid concentration 1281 ± 57 mg COD/L	0.218 gCOD/gCOD	She et al., 2020
Cheese whey	Mixed culture fermentation under different pH at 35°C	n/a	0.056 gCOD/gCOD under pH 5; 0.164 gCOD/gCOD under neutral pH; and 0.31 gCOD/gCOD under pH 10	Jankowska et al., 2017
Semi-synthetic milk processing wastewater	Mixed culture and pure culture fermentations under pH 10 at 35°C	743 mgCOD/L acetic acid by *C. aceticum;* 541 mgCOD/L acetic acid by mixed microbial culture	n/a	Atasoy et al., 2019a

Atasoy et al. (2019a) compared acetic acid production efficiency from monoculture and mixed culture during fermentation of milk processing wastewater under alkali pH. Higher acetic acid concentration was obtained from *C. aceticum* as 743 mg COD/L than the mixed culture as 541 mg COD/L (Atasoy et al., 2019a). The result of the current study showed that bioaugmentation of mixed culture with *C. aceticum* produced almost two times higher acetic acid concentration than monoculture fermentation. Talabardon et al. (2000) evaluated the acetic acid production from milk permeate under thermophilic (60°C) fermentation. Although many thermophilic acetogens are not able to ferment lactose, they investigated that *Clostridium thermolacticum* can convert lactate to acetate, ethanol, CO_2_ and H_2_ (Talabardon et al., 2000). From this point of view, our results stated that *C. aceticum* not only grew with cheese production wastewater as substrate but also it increased acetic acid production in mixed culture.

### Quantification of *Clostridium aceticum* During the Reactor Operation

The efficiency of the bioaugmentation was monitored *via* acetic acid production and the adaptation of *C. aceticum* in the mixed culture. For this reason, *C. aceticum* was quantified in every phase during the operation, as represented in Figure 2. The results showed that the average gene copy number of *C. aceticum* was 2.2×10^6^±1.5×10^3^ before bioaugmentation (Phase A), while it increased 4.6×10^6^±1.1×10^4^ during the application of bioaugmentation (Phase B). After the bioaugmentation was complete (Phase C), the gene copy number increased 5.3×10^6^±1.6×10^3^ on average. Based on the quantification of *C. aceticum*, the average copy gene number increased almost 2.5 times by bioaugmentation. The highest gene copy number was obtained on day 21 as 5.7×10^6^±1.2×10^2^. Nevertheless, as stated before, the highest acetic acid production was obtained at day 63.

Even though the main product of *C. aceticum* is acetic acid, there are several other products such as ethanol, butyric acid, and acetone etc., based on the metabolic pathway type (Jones et al., 2016). Arslan et al. (2019) investigated acetic acid and ethanol production by *C. aceticum* under acidic and alkali pH conditions (Arslan et al., 2019). Their results showed that C. *aceticum* had a fast biomass growth under pH 8 with 1.8 g acetic acid production in the first 44 h of the fermentation. Nevertheless, the biomass growth of *C. aceticum* gradually decreased when the pH reached acidic conditions. Additionally, they observed that *C. aceticum* converted ethanol to acetic acid in the solventogenic phase as a reverse reaction (Arslan et al., 2019). From the perspective of their results, the growth of *C. aceticum* in the mixed culture might be promoted by the alkali pH. The reverse reaction ability of *C. aceticum* could result in higher other acid types of production at *C. aceticum* in the bioaugmented reactor than the control reactor.

*Clostridium aceticum* has been studied mainly for autotrophic growth on carbon dioxide, carbon monoxide, and hydrogen gasses to produce acetic acid (Sim et al., 2007; Riegler et al., 2019). Nevertheless, the results of the current study revealed that *C. aceticum* adapted well to the mixed culture under high alkali pH (10) in carbon-rich substrate fermentation.

### Bacterial Community Profile

**FIGURE 3:**
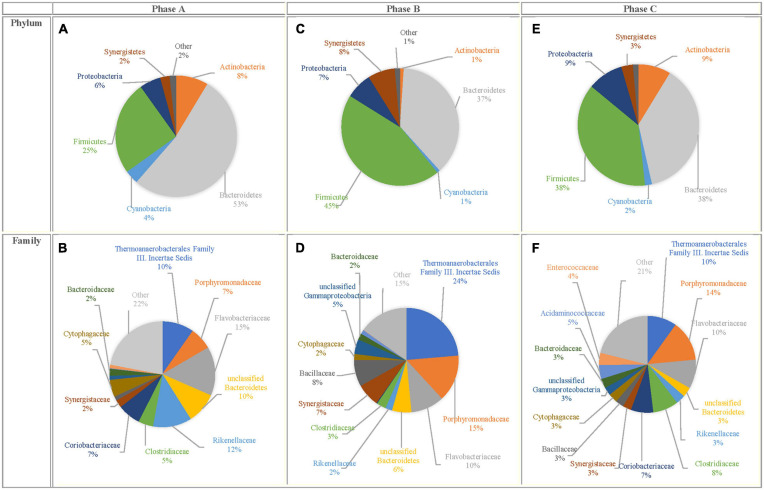
Bacterial community profile for Phase A, Phase B, and Phase C for phylum level **(A,C,E)** and family level **(B,D,F)**.

Besides the quantification of *C. aceticum*, the bacterial community profile in the bioaugmented reactor was analyzed. Analysis of the sequencing data for each phase (Phase A, B, and C) were presented as phylum and family levels in Figure 3. The results showed that despite the most dominant phylum, members were the same in each phase (Figures 3A,C,E). Their relative abundance varied depending on the bioaugmentation stage. The most dominant phylum was *Bacteroidetes* in Phase A (53 ± 12%), while its relative abundance decreased to 37 ± 9 and 38 ± 16% in Phase B and in Phase C, respectively. *Firmicutes* was the second most dominant phylum member in Phase A (25 ± 7%). The relative abundance of *Firmicutes* increased by bioaugmentation: it was 45 ± 4% in Phase B and 38 ± 11% in Phase C. In this regard, the bioaugmentation effect in each phase was more distinctly based on the relative abundance of family members (Figures 3B,D,F).

The bacterial community profile in the control reactor was presented in our recently published study (Atasoy and Cetecioglu, 2021). The results showed that the dominant phylum members changed through the reactor operation in the control reactor. *Firmicutes* (30 ± 3%) were dominant at the beginning of the reactor operation, while *Bacteroidetes* became dominant gradually, their relative abundance increased to 35 ± 11 and 49 ± 13% after almost 4 and 21 weeks of reactor operation, respectively (Atasoy and Cetecioglu, 2021).

The most dominant family members were *Flavobacteriaceae* (15 ± 6%) in Phase A; *Thermoanaerobacterales Family III*. *Incertae sedis* (24 ± 6%) in Phase B and *Porphyromonadaceae* (14 ± 7%) in Phase C, respectively in the bioaugmented reactor. On the other hand, the most dominant family members in the control reactor were *Porphyromonadaceae* (12 ± 2%), *Coriobacteriaceae* (8 ± 3%), and *Flavobacteriaceae* (8 ± 5%) at the beginning of the reactor operation. After 21 weeks, *Porphyromonadaceae* (27 ± 12%), *Bacteroidaceae* (17 ± 4%), and *Veillonellaceae* (11 ± 3%) became dominant in the control reactor (Atasoy and Cetecioglu, 2021).

*Thermoanaerobacterales Family III*. *Incertae sedis* belongs to the *Firmicutes* phylum, main fermentation products are acetate, succinate, ethanol and formate (Gries et al., 2019). The results revealed that the relative abundance of *Thermoanaerobacterales Family III. Incertae sedis* increased simultaneously with the gene copy number of *C. aceticum* during bioaugmentation. From this point of view, despite that, there is no study in the literature about the relations between species of the *Thermoanaerobacterales Family III*. *Incertae sedis* and *C. aceticum*, the results of the current study suggest that there might be a syntropy for acetic acid production.

The relative abundance of *Porphyromonadaceae*, which is a family member of *Bacteroidetes* phylum, increased by bioaugmentation. Since *Porphyromonadaceae* is mainly responsible for propionic acid production (Rios-Covian et al., 2017), an increase in propionic acid production during and after bioaugmentation might link with the high abundance of *Porphyromonadaceae.* Furthermore, *Porphyromonadaceae* (27 ± 12%) was the most dominant family member in the control reactor at the end of the reactor operation (Atasoy and Cetecioglu, 2021).

The relative abundance of *Rikenellaceae* from the phylum of *Bacteroidetes*, dramatically decreased by bioaugmentation: it was 12 ± 3% in Phase A; 2 ± 0.7% in Phase B, and 3 ± 1.02% in Phase C. Apart from the effects of bioaugmentation, the relative abundance of *Rikenellaceae* decreased by the retention time in anaerobic digesters (Nakasaki et al., 2020). *Rikenellaceae* is a well-known species in anaerobic digesters (Koo et al., 2019; Nakasaki et al., 2020; Schwan et al., 2020) and participates in easily degradable compounds (i.e., glycerol) degradation to acetic acid in fermentation (Nakasaki et al., 2020). In the current study, the decreasing relative abundance of *Rikenellaceae* in Phase B and Phase C might be caused by either rapid consumption of readily biodegradable compounds or competing with *C. aceticum* and members of the *Rikenellaceae* family.

### Volatile Fatty Acid Composition Shift at the Bioaugmented Reactor

**FIGURE 4:**
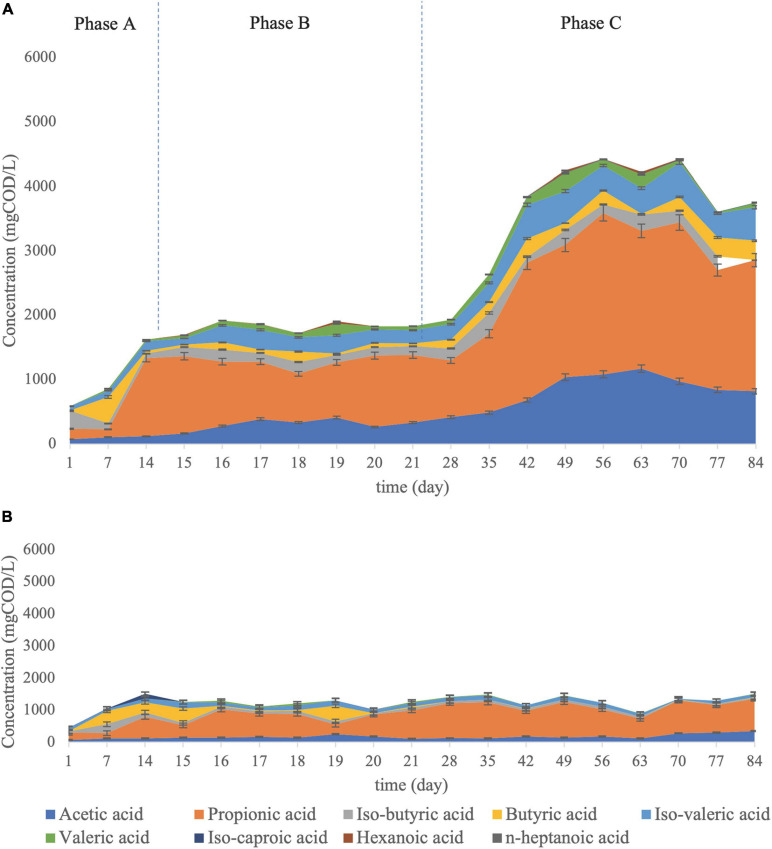
VFA composition **(A)** in the *C. aceticum* bioaugmented reactor at Phase A: before bioaugmentation; Phase B: during bioaugmentation, and Phase C: after bioaugmentation and **(B)** in the control reactor.

The main aim of mixed culture bioaugmentation with *C. aceticum* was to enhance acetic acid production in the VFA mixture. As stated before, acetic acid concentration was increased by bioaugmentation by *C. aceticum*: the average acetic acid percentage in the VFA mixture was 8 ± 0.2% in Phase A, 15 ± 4% in Phase B and 22 ± 5% in Phase C (Figure 4A). Interestingly, the results stated that the dominant acid type, which was propionic acid, did not change by bioaugmentation. Although *C. aceticum* is not responsible for propionic acid production according to its metabolic pathway (Alexander and Weuster-Botz, 2017; Arslan et al., 2019), propionic acid production was enhanced by bioaugmentation of *C. aceticum*. The average percentage propionic acid concentration in the VFA mixture was 34 ± 23% in Phase A, 55 ± 13% in Phase B, and 49 ± 9% in Phase C. The dominant acid type was propionic acid in the control reactor during operation (Figure 4B). The acid composition in the control reactor as an average was: 59 ± 12% propionic acid, 13 ± 7% acetic acid, 8 ± 3% isovaleric acid, 7 ± 2% butyric acid, and 6 ± 3% isobutyric acid. The results stated that despite the propionic acid was dominant at both reactors, the bioaugmentation of *C. aceticum* suppressed the propionic acid percentage in the VFA mixture, while it increased the ratio of acetic acid.

The manipulation of the operational conditions in mixed culture fermentation can affect the acid composition (Lu et al., 2011). Nevertheless, control of the manipulation mechanism depends on thermodynamic and metabolic principles: the product mixture is specified by thermodynamic constraints and enzyme availability in pure culture fermentation, while, it is more complicated in mixed culture fermentation because of the wide range of metabolic activities and energetic considerations (Mohd-Zaki et al., 2016). Many studies have been conducted to identify metabolic flux and energy conversions in mixed culture fermentation (Lee et al., 2008; Hoelzle et al., 2014; De Vrieze and Verstraete, 2016; Cetecioglu et al., 2019). Lee et al. (2008) evaluated H_2_ production from glucose fermentation under several pH conditions, thermodynamically (Lee et al., 2008). Based on their calculations, the standard Gibbs Free Energy (ΔG°)′ for acetic acid and propionic acid production *via* glucose fermentation under pH 10 stated that acetic acid production has −1,14 kJ/e^–^; propionic acid production has −6,78 kJ/e^–^. Though thermodynamic calculations in fermentation depend on many parameters such as pH, temperature, substrate composition, therefore available electron acceptors, etc., the rough estimate would be an explanation for high propionic acid production in the bioaugmented reactor since propionic acid production provides more energy than acetic acid production.

Besides acetic and propionic acid production, other acid type production during the operation were observed: the average acid composition during Phase C was composed of 11 ± 8% of iso-valeric acid, 6 ± 4% of valeric acid, 5 ± 4% of iso-butyric acid, and 5 ± 3.2% of butyric acid in addition to acetic acid and propionic acid. Therefore, the bioaugmentation of *C. aceticum* enhanced not only acetic acid production but also increased propionic acid production indirectly and other acid productions (Alexander and Weuster-Botz, 2017). Nevertheless, the bioaugmentation of mixed culture by *C. butyricum* showed that the dominant acid type (propionic acid) was shifted to butyric acid by bioaugmentation (Atasoy and Cetecioglu, 2020). In addition, their results stated that bioaugmentation of *C. butyricum* did not only change dominant acid type but also significantly affect the VFA composition as well: propionic acid was decreased (from 60 ± 12 to 30 ± 17%) whereas, acetic (from 12 ± 7 to 20 ± 11%), butyric (from 21 ± 9 to 35 ± 4%), and valeric (from 8 ± 4 to 15 ± 5%) acids were increased by bioaugmentation (Atasoy and Cetecioglu, 2020). Therefore, despite pH (Atasoy et al., 2019b; Eryildiz et al., 2020), substrate type (Jankowska et al., 2017; Ma et al., 2017), and temperature (Jiang et al., 2013; Vanwonterghem et al., 2015) affected the VFA composition, the current results showed that type of monoculture in bioaugmentation is also one of the important parameters to effect acid type in fermentation.

### Total VFA Production at the Bioaugmented Reactor

The total VFA production in the bioaugmented reactor was 1204 ± 340 mg COD/L (0.67 gCOD/gCOD yield) at Phase A, 1878 ± 338 mg COD/L (0.79 gCOD/gCOD yield) at Phase B, and 4166 ± 988 mg COD/L (0,85 gCOD/gCOD yield) at Phase C. Also, the highest total VFA production was obtained at day 70th as 5493 ± 36 mg COD/L with a 0,98 gCOD/gCOD production yield. In the control reactor, the average total VFA concentration was 1254 ± 11 mg COD/L with 0.68 ± 0.02 gCOD/gCOD yield. Thus, based on the total VFA concentration in the bioaugmented and the control reactor, the average concentration increased 3.3 times, whereas the maximum concentration increased 5 times.

The efficiency of VFA production *via* fermentation depends on several operational and environmental conditions. Jankowska et al. (2017) showed the importance of substrate type on VFA production (Jankowska et al., 2017). Their results stated that the highest VFA was obtained from microalgae biomass (0.83 gVFA/gSCOD), maize silage (0.78 gVFA/gSCOD), and cheese whey (0.71 gVFA/gSCOD), respectively, under initial alkaline pH conditions. Moretto et al. (2019) investigated the optimized conditions for VFA production under mesophilic and thermophilic conditions with different pH values by using a batch reactor and continuously stirred tank reactor (CSTR) from urban waste fermentation (Moretto et al., 2019). Their results showed that the batch reactor and CSTR produced almost the same VFA concentration with thermal pretreatment under pH 9 (41 ± 2 gCOD/L for batch; 39 gCOD/L for CSTR). The pH did not affect VFA concentration (pH 9: 30 ± 2 gCOD/L; pH 7: 27.5 ± 2 gCOD/L) as well (Moretto et al., 2019). In addition, Atasoy et al. (2019b) confirmed that the pH had almost no effect on VFA production yield. From the point of parameters that affect VFA production, the results of the current study stated that the VFA production depends on the type of bacterial strain as well as their interactions with each other and their environment.

### The Correlation Analysis Between VFA Composition and Quantity of *C. aceticum*

The concentration of each acid type and quantity of *C. aceticum* was correlated during fermentation (Phase A, B, and C) to investigate their mutual effect on each other, the results are shown in Table 2. The correlation analysis results stated that acetic acid production positively correlated with total VFA (0.898), iso-valeric acid (0.866), propionic acid (0.789), and iso-hexanoic acid (0.720) production at a 0.01 significance level as well as the gene copy number of *C. aceticum* (0.553) at 0.05 significance level. Nevertheless, the bioaugmentation of *C. aceticum* resulted in higher propionic acid production in all phases. There was no correlation between propionic acid production and the gene copy number of *C. aceticum*. However, the total VFA production was correlated (0.490 at 0.05 significance level) with *C. aceticum*. Therefore, the correlation analysis of *C. aceticum* bioaugmentation reactor data might be explained as *C. aceticum* enhanced propionic acid production indirectly.

**TABLE 2:** Correlation coefficients between the quantification of *C. aceticum* with VFA composition after bioaugmentation.

	**Acetic**	**Propionic**	**Iso-butyric**	**Butyric**	**Iso-valeric**	**Valeric**	**Iso hexanoic**	**Hexanoic**	**n-heptanic**	**Total**	***C.aceticum***
	**acid**	**acid**	**acid**	**acid**	**acid**	**acid**	**acid**	**acid**	**acid**	**VFA**	**copy number**
Acetic acid	1	0.789**	0.312	0.012	0.866**	0.542*	0.720**	0.424	0.551*	0.898**	0.553*
Propionic acid	0.789**	1	0.226	0.013	0.878**	0.433	0.770**	0.383	0.197	0.968**	0.437
Iso-butyric acid	0.312	0.226	1	0.476*	0.471*	–0.095	0.583**	–0.217	0.426	0.361	0.194
Butyric acid	0.012	0.013	0.476*	1	0.073	–0.402	0.403	−0.468*	0.058	0.109	–0.33
Iso-valeric acid	0.866**	0.878**	0.471*	0.073	1	0.489*	0.729**	0.277	0.406	0.943**	0.646**
Valeric acid	0.542*	0.433	–0.095	–0.402	0.489*	1	–0.023	0.837**	0.329	0.483*	0.42
Iso hexanoic acid	0.720**	0.770**	0.583**	0.403	0.729**	–0.023	1	0.01	0.292	0.816**	0.233
Hexanoic acid	0.424	0.383	–0.217	−0.468*	0.277	0.837**	0.01	1	0.151	0.379	0.173
n-heptanoic acid	0.551*	0.197	0.426	0.058	0.406	0.329	0.292	0.151	1	0.355	0.177
Total VFA	0.898**	0.968**	0.361	0.109	0.943**	0.483*	0.816**	0.379	0.355	1	0.490*
*C. aceticum* copy number	0.553*	0.437	0.194	–0.33	0.646**	0.42	0.233	0.173	0.177	0.490*	1

**Correlation is significant at the 0.01 level (2-tailed); *Correlation is significant at the 0.05 level (2-tailed).

### Cycle Analysis in the Bioaugmented Reactor

**FIGURE 5:**
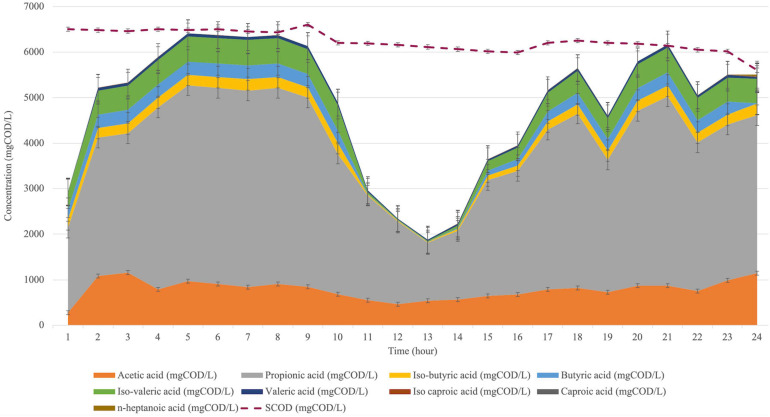
VFA composition shift during a cycle analysis at the bioaugmented reactor for 24 h.

The acid composition was observed during a cycle (24-hour) to investigate the acid shift. Therefore, the cycle analysis was conducted after seven days of bioaugmentation application (day 28). The acid shift during a cycle for the bioaugmented reactor is represented in Figure 5.

The cycle analysis of *C. aceticum* in the bioaugmented reactor showed that almost all acid types were increased to the peak point in the first 5 h, then it was stable until the 9th hour. From the 13th hour, the production was increased again until at the end of the cycle. Mainly, acetic acid production in the 1st hour was 279 ± 17 mg COD/L, then it increased in the first 3 h to 1157 ± 29 mg COD/L. Following this, the production was almost stable in the next 14 h; afterwards, it reached 1148 ± 36 mg COD/L at the 24th hour. Propionic acid, which was the dominant acid type during a cycle, started from 1859 ± 204 mg COD/L in the 1st hour. It reached 3465 ± 108 mg COD/L at the end of the cycle (24th hour), although the highest propionic acid concentration was between the 5th and 9th hours.

The sCOD concentration from the 1st hour to the 24th hour (Figure 5) showed that the sCOD reduction was negligible. Nevertheless, the reason for the drop in total VFA production between the 11th and 17th hours could be explained by CO_2_ production. On the other hand, as described previously, the metabolic pathway of *C. aceticum* has reversible reactions from acetic acid to ethanol production as well as vice versa (Grimalt-Alemany et al., 2018). Therefore, the results might be explained by chain elongation (De Groof et al., 2019) from short-chain fatty acids (VFA) to medium or large chain fatty acids as well as ethanol or CO_2_ production after the 16th hour. Following this, the reversible reactions might have increased VFA production again from the 14th hour. The cycle analysis stated that the highest VFA production, as well as acetic acid concentration, could be obtained in the first 6 h of the cycle, shortening the retention time or reduction of the reactor volume. In addition, cycle analysis was also conducted in the control reactor. The results showed that 12 h would be sufficient as a cycle duration to achieve a similar VFA composition and production efficiency in the control reactor (Atasoy and Cetecioglu, 2020).

## Conclusion

In this study, mixed microbial culture was bioaugmented by *C. aceticum* to obtain an acetic acid dominant VFA mixture. Despite the fact that acetic acid concentration was increased by bioaugmentation (almost 10-fold compared to the control reactor), the dominance of the propionic acid in the VFA mixture did not change. However, the effects of the bioaugmentation indicate certain unknown syntrophic relations and corresponding metabolic pathways. It is crucial to gain a deeper understanding of bioaugmentation’s effects on the microbial community, particularly to establish its profile and the functions and interactions in the mixed culture. Furthermore, the identification of metabolic networks for acid production is crucial for a comprehensive view of bioaugmentation effects.

## Data Availability Statement

The datasets presented in this study can be found in online repositories. The names of the repository/repositories and accession number(s) can be found below: https://www.ncbi.nlm.nih.gov/, PRJNA667606.

## Author Contributions

MA: conceptualization, methodology, formal analysis, investigation, data curation, writing – original draft, and visualization. ZC: conceptualization, writing – review and editing, and supervision. Both authors contributed to the article and approved the submitted version.

## Conflict of Interest

The authors declare that the research was conducted in the absence of any commercial or financial relationships that could be construed as a potential conflict of interest.

## Publisher’s Note

All claims expressed in this article are solely those of the authors and do not necessarily represent those of their affiliated organizations, or those of the publisher, the editors and the reviewers. Any product that may be evaluated in this article, or claim that may be made by its manufacturer, is not guaranteed or endorsed by the publisher.
